# All-nanophotonic NEMS biosensor on a chip

**DOI:** 10.1038/srep10968

**Published:** 2015-06-04

**Authors:** Dmitry Yu. Fedyanin, Yury V. Stebunov

**Affiliations:** 1 Laboratory of Nanooptics and Plasmonics, Moscow Institute of Physics and Technology, Dolgoprudny, 141707 Russian Federation

## Abstract

Integrated chemical and biological sensors give advantages in cost, size and weight reduction and open new prospects for parallel monitoring and analysis. Biosensors based on nanoelectromechanical systems (NEMS) are the most attractive candidates for the integrated platform. However, actuation and transduction techniques (e.g. electrostatic, magnetomotive, thermal or piezoelectric) limit their operation to laboratory conditions. All-optical approach gives the possibility to overcome this problem, nevertheless, the existing schemes are either fundamentally macroscopic or excessively complicated and expensive in mass production. Here we propose a novel scheme of extremely compact NEMS biosensor monolithically integrated on a chip with all-nanophotonic transduction and actuation. It consists of the nanophotonic waveguide and the nanobeam cantilever placed above the waveguide, both fabricated in the same CMOS-compatible process. Being in the near field of the strongly confined photonic or plasmonic mode, cantilever is efficiently actuated and its response is directly read out using the same waveguide, which results in a very high sensitivity and capability of single-molecule detection even in atmosphere.

Advances in fabrication technologies make it possible to manufacture nanoscale chemical and biological sensors and integrate them in a wide range of different devices. This does not only decrease the size but also improves the resolution, reduces the price and opens the way to parallel monitoring and analysis on a chip. Nanoelectromechanical systems (NEMS) are considered to be the most attractive platform. NEMS biosensors are essentially compact, guarantee extremely high sensitivity with a detection down to the single molecule level and demonstrate a very broad range of possible applications from mass spectrometry to medical diagnostics^1^,^2^,^3^. Specific surfaces deposited on NEMS can adsorb detecting agents in vacuum, gaseous and fluid environments, which changes the oscillating mass and shifts the resonant frequency of mechanical oscillations. However, practical implementation of NEMS sensors is hindered by actuation and transduction schemes. Mechanical oscillations can be excited and detected using electrostatic^4^,^5^, magnetomotive^6^, thermal^7^,^8^,^9^, piezoresistive^10^ and piezoelectric^11^ techniques, but all these methods have significant disadvantages^3^. For the magnetomotive actuation, a very high magnetic field is needed, which increases the device size and its price and makes it difficult to operate^6^. Electrostatic schemes are rather inefficient at high resonant frequencies and small dimensions of NEMS^3^,^12^. Thermal actuation and piezoresistive transduction induce heating, which changes the kinetics of biomolecular reactions and consequently decreases the accuracy of measurements. Furthermore, the operating frequency is significantly limited by the finite thermal conductance of the cantilever^15^,^16^. Piezoelectric actuation at high resonant frequencies requires complex multilayer nanostructures with a thin piezolelectric layer. This impairs mechanical characteristics of the cantilever and affects chemical properties of its surface^17^. Finally, the above mentioned techniques introduce additional damping^13^, which reduces the quality factor of NEMS, and their application in liquid and high pressure gas environments is seriously restrained by high leakage currents^18^. All-optical approach can eliminate these drawbacks and simplify the design of NEMS biosensors.

Optical actuation and detection schemes based on a laser beam have been used for years^19^,^20^,^21^, but, being fundamentally macroscopic, they can hardly be a part of an integrated biosensor platform. Optical fibers confine light on the optical-wavelength scale and make the devices microscopic^22^,^23^. This drastically increases sensitivity and accuracy of measurements. In spite of these advantages, such biosensors are quite bulky and expensive in mass production, which is dictated by the necessity to precisely position and align the fiber at a submicrometer distance from the NEMS. To develop a truly integrated NEMS biosensor, optical readout and actuation schemes should be fabricated directly on a chip, which can be achieved with planar waveguide technologies. Efficient readout of nanomechanical motion could be realized using coupling between guided and radiation modes of a waveguide^24^,^25^,^26^,^27^, coupling between a waveguide and an optical resonator^28^,^29^,^30^, end-to-end coupling of waveguides^31^,^32^ and side-coupling between a cantilever and a waveguide^33^,^34^. Such configurations provide high displacement sensitivity and can be fabricated on a chip. However, their implementation is significantly restricted by the excessive complexity of fabrication processes.

Here, we present a novel type of strongly integrated on-chip NEMS biosensors with all-nanophotonic transduction and actuation. By placing a nanobeam cantilever at a subwavelength distance above a silicon-on-insulator (SOI) waveguide, one can use this waveguide for optical transduction and actuation of the cantilever. This gives a possibility to simplify the NEMS biosensor design, reduce its size and integrate it on a chip. At the same time, the proposed scheme shows high sensitivity and efficiency thanks to the near-field optomechanical interaction. Moreover, performance characteristics can be further improved by implementing CMOS-compatible silicon nitride photonic waveguides and copper plasmonic waveguides. The latter exhibit the highest transduction and actuation efficiency and are capable of single-molecule detection even in atmosphere.

## Results

### All-nanophotonic NEMS biosensor design and operating principle

**Figure 1:**
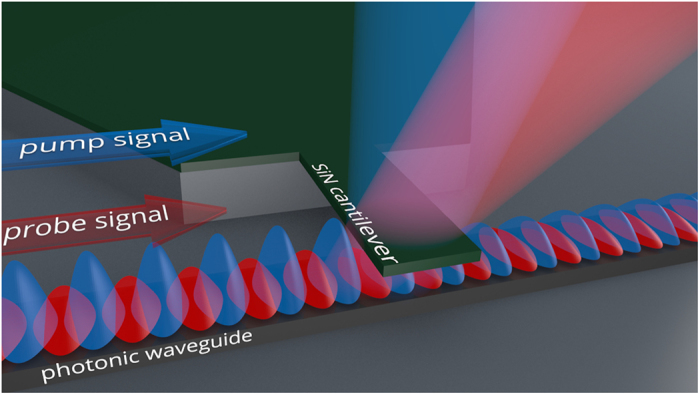
Schematic view of the highly integrated all-nanophotonic NEMS biosensor. Nanobeam cantilever is suspended above the photonic waveguide at a distance ranging from a few tens to a few hundreds of nanometers and is in the near-field of the optical mode of the waveguide. Pump optical signal excited at a light wavelength λ_1_ and sinusoidally modulated at a frequency *f*_M_ actuates the cantilever. At the same time, the power of the continuous wave probe signal excited at a light wavelength λ_2_ and propagating along the same waveguide is controlled by the vibrating nanobeam, which gives a possibility to gauge the amplitude of mechanical oscillations. By doing the scan of the modulation frequency *f*_M_, one can measure the decrease in the resonant frequency of the cantilever and determine the mass of adsorbed molecules.

Figure 1 shows the proposed configuration of the NEMS biosensor with all-nanophotonic transduction and actuation. It consists of only two basic elements: a nanophotonic waveguide and a free-oscillating cantilever aligned approximately perpendicular to the waveguide and placed above it at a small distance ranging from a few tens to a few hundreds of nanometers. In this case, the cantilever is in the waveguide near field, which induces mutual interaction between guided optical modes and the cantilever. First of all, the cantilever is a fairly large scatter for optical modes of the waveguide and, as the distance between the cantilever and the waveguide decreases, more radiation is scattered to free space and, consequently, less power is transmitted through the waveguide section with the cantilever. Accordingly, the oscillating cantilever modulates the intensity of the probe optical signal at a frequency of mechanical oscillations. Detecting molecules adsorbed on the surface of the cantilever change the resonant frequency of mechanical oscillations (dynamic regime) or create a surface tension resulting in cantilever bending (static regime). Both the magnitude of the transmitted optical signal and the frequency of modulation can be precisely measured with a photodetector and report the mass of adsorbed molecules and the adsorption rate, which forms an exceptionally efficient and accurate transduction scheme. Secondly, the electric field amplitude of the guided optical mode rapidly decreases with the distance from the waveguide core and the cantilever experiences a ponderomotive force, which is proportional to the gradient of the squared electric field. Thanks to the extremely high mode localization in nanophotonic waveguides, even a weak pump signal provides a few nanometers displacement of the cantilever. Being excited at a wavelength different from that of the probe signal and modulated at the resonant frequency of mechanical oscillations, such a low power pump signal actuates the NEMS without any effect on the transduction scheme. In contrast to electric and magnetic actuation techniques, the photonic approach does not introduce inherent limitations in the modulation bandwidth except that the electro-optic modulator is typically limited by a frequency of the order of 1 GHz, which is much higher than the resonant frequency of mechanical oscillations.

Mechanical cantilever can be fabricated from different materials and have different shapes, but for on-chip integration, compatibility with conventional silicon CMOS fabrication processes and planar technologies is essential. This makes the nanobeam one of the best candidates, since it is very small in size, allows operation at high oscillation frequencies and is easily fabricated. Silicon seems to be the best material for the cantilever in photonic schemes owing to the high refractive index, but gas and biosensors typically operate in aggressive environments and chemical reactions (such as oxidation) on the silicon surface are practically unavoidable. They alter elasticity properties of the cantilever and change the oscillating mass along with the gas or biological molecules adsorbed on the cantilever, which significantly deteriorates the accuracy of measurements. In this regard, silicon nitride (SiN) is much preferred thanks to the high chemical stability and roughly the same mechanical properties as that of silicon. Small SiN beam with dimensions of 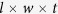 clamped at one end and free at the other exhibits the lowest mechanical resonance at the frequency^35^,^36^


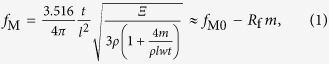


where *Ξ* = 290 GPa and *ρ* = 3 g/cm^3^ 37 are the Young’s modulus and mass density of the SiN beam, respectively, *f*_M0_ is the resonant frequency of the cantilever, *m* is the mass of molecules adsorbed at the end of the cantilever and *R*_f_ is the mass responsivity. For the SiN beam dimensions of 5 μm × 1 μm × 90 nm, the quality factor of the resonance is about *Q*_M _= 3500^19^, *f*_M0_ = 5.72 MHz and 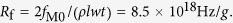


Mode properties of the photonic waveguide are not as sensitive to ultrathin oxide layers on the silicon surface as that of mechanical ones of the cantilever, and the nanophotonic waveguide can be realized on a standard SOI platform. Thus, 200 nm thick silicon strip on a silicon dioxide (SiO_2_) layer confines light on the nanoscale and the penetration depth of the only guided TE_0_ mode into the air is *ζ*_top_ = 96 nm at λ = 1.55 μm, which is small enough for efficient transduction and actuation.

### Photonic transduction of the nanocantilever

Optical mode propagating in the SOI waveguide passes through the section, where the SiN beam is located above the waveguide, and is partially reflected back, but mostly scattered into free space. When the distance *h* between the nanobeam and the waveguide is much larger than the penetration depth *ζ*_top_ of the electromagnetic field of the optical mode into air or vacuum, the transmission coefficient *T* is nearly equal to unity and there is no practical interaction between the cantilever and the optical mode. As the distance *h* decreases, the SiN nanobeam changes significantly the refractive index profile of the waveguide and induce scattering. This can be treated as coupling between guided and radiation modes of the unperturbed waveguide^38^,^39^,^40^ and can be expressed as


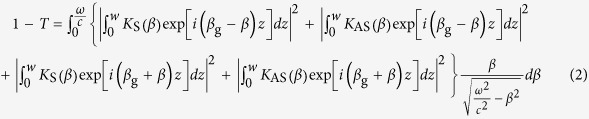


where *β*_g_ is the wavenumber of the guided mode and *K*_S_(*β*) and *K*_AS_(*β*) are the coupling coefficient between the guided mode and symmetric and anti-symmetric radiation modes, respectively:


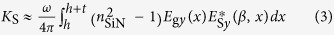



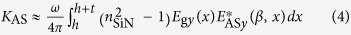


In the above expressions, *n*_SiN _= 1.98^41^ is the refractive index of SiN, *h* is the separation distance between the cantilever and the waveguide surface, *t* is the thickness of the cantilever beam, *E*_g*y*_(*x*), *E*_S*y*_(*β,x*) and *E*_AS*y*_(*β,x*) are the normalized^39^ transverse electric field amplitudes of the guided mode, symmetric radiation mode with the wavenumber *β* and anti-symmetric radiation mode with the wavenumber *β*, respectively. Equations (2) and (3) clearly show that the scattered power is approximately proportional to the square of electric-field amplitude at the position of the cantilever |*E*_gy_(*h*)|^2^ and decreases with the distance *h* as 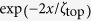. Accordingly, the oscillating beam modulates the total transmission with a modulation depth of 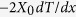, where *X*_0_ is the amplitude of mechanical oscillations.

**Figure 2:**
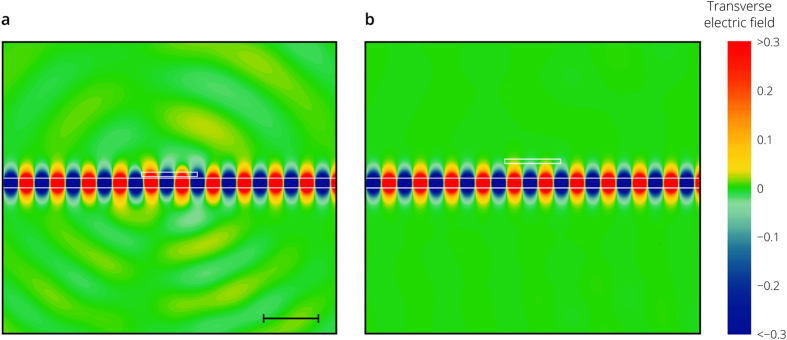
Field distribution for the fundamental TE mode of the SOI waveguide passing through the section with the cantilever. Transverse electric field distribution of the fundamental photonic mode guided by the 200 nm thick SOI waveguide and transmitted through the section with the SiN cantilever placed above the waveguide for two positions of the cantilever: 30 nm (panel a) and 300 nm (panel b) above the silicon waveguide. The scale bar is 1 μm and the light wavelength is 1.55 μm. At a separation distance of 30 nm between the waveguide and the cantilever, probe signal loses 0.35% of its power. As the distance between the cantilever beam and the waveguide increases, less power is scattered out and reflected back and, at a distance of 300 nm, the optical loss does not exceed 0.001%.

**Figure 3:**
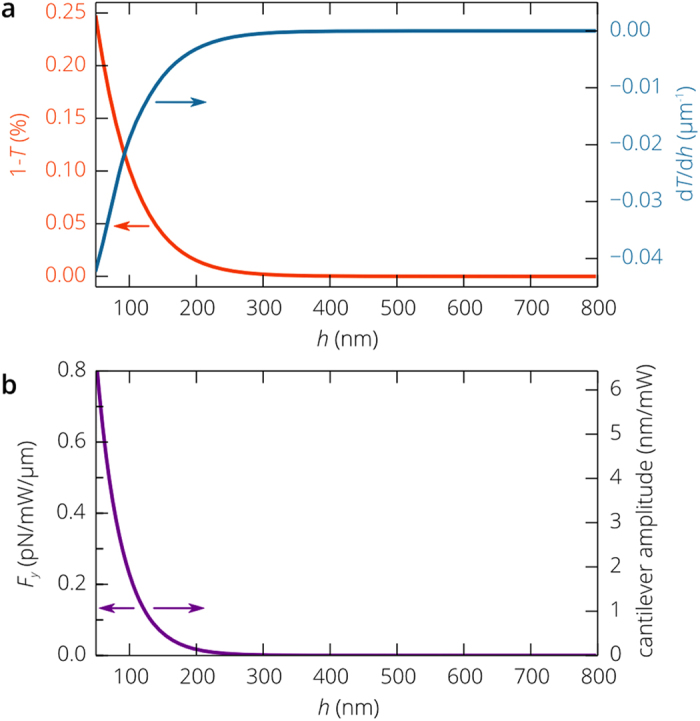
Transduction and actuation of the SiN cantilever with the SOI waveguide. (**a**) Simulated transmittance *T* of the probe optical signal through the waveguide section with the cantilever (see Fig. 2) and its derivative *dT*/*dh* as a function of the cantilever position. (**b**) Optical force generated on the cantilever by the continuous wave pump signal per unit power of the pump signal per unit length of the cantilever beam at a light wavelength of 1.31 μm and the amplitude of mechanical oscillations induced by the fully modulated pump optical signal propagating along the 1 μm wide silicon waveguide.

Figure 2 shows the distribution of the transverse electric field for two different positions of the cantilever simulated using the finite element method (see Methods). As the distance *h* decreases, more energy of the guided optical mode is scattered by the cantilever both forward and backward (Fig. 2), which decreases the total transmission. The dependence of the transmitted power and the linear displacement detection responsivity *R*_X _= –d*T*/d*x* on the cantilever position is presented in Fig. 3a. The simulation results are in a good agreement with the prediction of the coupled mode theory. Despite that only 1 – *T* = 0.25% of the mode power is lost at *h* = 50 nm, the responsivity *R*_X_ = 0.04 μm^−1^ is higher than that of the fiber based systems^23^, which creates the backbone for the realization of extremely compact and efficient gas and biosensors integrated on a chip.

### Optomechanical actuation of the nanocantilever

The guided optical mode of the SOI waveguide is highly confined to the silicon core and its electric field decays exponentially with the distance from the silicon surface. The penetration depth into air is less than 100 nm at telecom wavelengths, which creates an extremely strong field gradient in the near field of the waveguide. Electric field polarizes the nanobeam and the positive and negative charges experience noticeably different forces due to the field gradient. Eventually, this results in a sufficiently large ponderomotive force acting on the cantilever, which can be exploited for actuation.

Net optical force ***F*** generated on the cantilever includes the scattering force and the dominant gradient force and can be calculated by integrating the Maxwell’s stress tensor 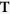 around the surface *S* encapsulating the nanobeam^42^:


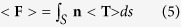


Here 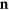 is the unit normal vector to the surface and the notation <…> represents averaging over the period *c*/*λ* of optical oscillations. Continuous wave pump signal with a power of 1 mW at a light wavelength of 1.3 μm propagating along the 1 μm wide SOI waveguide produces a force of about 0.8 pN acting on the cantilever (Fig. 3b). The fully modulated pump signal induces cantilever oscillations with the amplitude


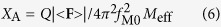


In vacuum and low-pressure gases, the amplitude *X*_A_ exceeds 7.0 nm (Fig. 3b), which is easily measured thanks to the high linear displacement detection responsivity of the proposed readout scheme.

### Sensitivity

Sensitivity of the proposed sensor is fundamentally limited by the thermomechanical noise of the cantilever and the noise of the probe optical signal. Thermal force acting on the cantilever has a random phase and a white spectrum with the spectral density 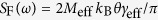 where *θ* is the temperature of the cantilever, *M*_eff_ is the effective mass of the cantilever and *γ*_eff_ = 2π*f*_M0_/*Q* is the effective damping constant. Spectral density of the corresponding random cantilever displacement is expressed as:


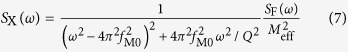


In resonance, *S*_X_(ω) increases up to *S*_X0_ = 5.9 ×10^−6^ nm^2^/Hz at room temperature, which eventually is converted to the relative intensity noise (RIN) at the photodetector *RIN*_X_ = S_X0_*R*_X_^2^. When the SiN nanobeam is 50 nm above the silicon waveguide, *RIN*_X_ = –150 dB/Hz exceeds the inherent RIN of the probe optical signal, which is below –120 dB/Hz for low cost semiconductor laser diodes and is in the range between –180 and –150 dB/Hz for lasers used in telecommunications. At the same time, we should note that the root mean square (r.m.s.) fluctuation amplitude of the cantilever is only 0.97 Å, which is much smaller than the amplitude of the oscillations induced by the 1 mW pump signal if the cantilever is separated from the waveguide by a distance shorter than 150 nm (Fig. 3b). Thus, the all-nanophotonic transduction-actuation scheme does not practically limit the sensitivity of the device, which is mostly determined by mechanical properties of the cantilever and can be evaluated as^43^


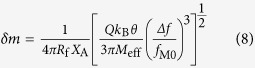


where Δ*f* is the measurement bandwidth, which is of the order of the inverse measurement time 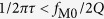
44. Accordingly, the considered SiN cantilever placed 50 nm above the silicon waveguide and excited by the fully modulated 1 mW pump signal demonstrates a sensitivity of about δ*m* = 4 kDa at a measurement bandwidth of 100 Hz, and the sensitivity is improved up to δ*m *= 130 Da as Δ*f* is decreased down to 10 Hz.

### Improvement of the all-nanophotonic gas and biosensor

The proposed transduction scheme based on a silicon waveguide and a SiN cantilever demonstrates a high linear displacement detection responsivity of more than 0.04 μm^−1^. At the same time, end-to-end coupled nanophotonic waveguide cantilevers can give *R*_X_ of about 1 μm^−1^ 31. The reason for such a high responsivity is that transmission through the gap separating two waveguide cantilevers is very sensitive to the relative position of the waveguide ends thanks to the high mode confinement^31^. In the case of the mechanical cantilever suspended above the waveguide (Fig. 1), the dependence of the transmission coefficient *T* on the separation distance between the cantilever and the waveguide is the result of the interplay between two opposite effects. The power loss of the guided mode 1 – *T* is proportional to the square of the normalized electric-field amplitude of the guided mode at the position of the cantilever *E*g*y*(*h*) (see equation (2)). First, this means that 1 – *T* decreases with the distance *h* approximately as 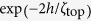 and, consequently, 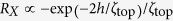. In other words, the higher mode confinement, which is characterized by 1/*ζ*_top_, the higher the linear displacement responsivity *R*_X_. Second, *R*_X_ also depends on the normalized amplitude of the electric field at *x* = *h*, which can be calculated from the equation 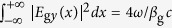
39. The same interplay between the magnitude of the normalized electric field at *x* = *h* and the field gradient is evident in actuation, which is governed by the ponderomotive optical force. If the refractive index contrast between the waveguide core and the claddings is very high, the energy flows mostly in the waveguide core and the amplitude of the electric field in the claddings is much smaller than that in the center of the core, while 1/*ζ*_top_ can be very large. In the opposite case of the low refractive index contrast, the power flow is spread out over a wide area in the waveguide cross-section and both the normalized field amplitude at the position of the cantilever and the inverse penetration depth into the cladding are very small resulting in a very low responsivity. This demonstrates that strong mode confinement is not a cornerstone for high responsivity and efficient actuation.

**Figure 4:**
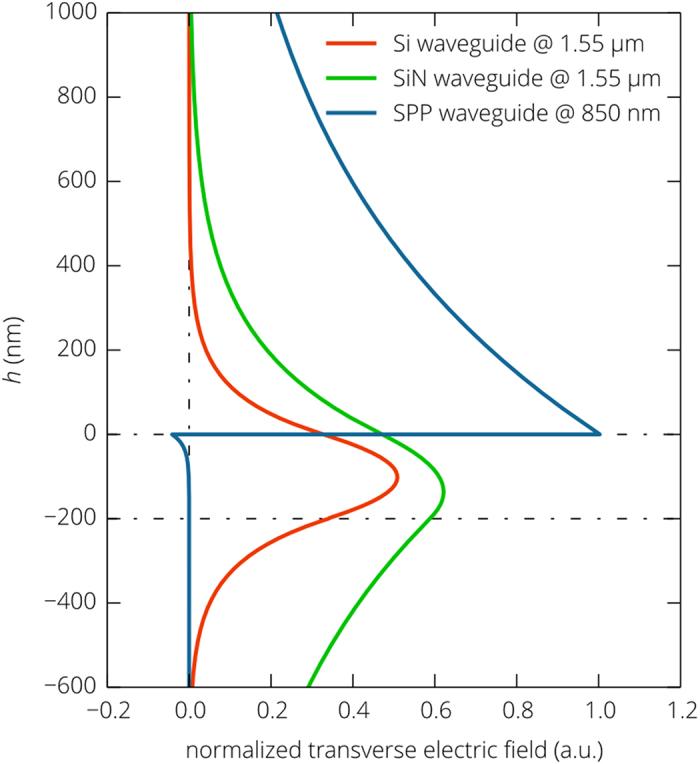
Normalized field distributions for the fundamental modes of photonic and plasmonic waveguides. Transverse electric fields for the fundamental TE mode of the Si and SiN photonic waveguides and for the fundamental TM mode of the SPP waveguide. The fields of the modes are normalized to carry the same power. Penetration depth of the optical mode in the SiN waveguide is twice as large as that in the Si one, the intensity of the SPP mode near the metal surface is much higher and the electrical field decays slower than that of the photonics mode of the Si and SiN waveguide.

**Figure 5:**
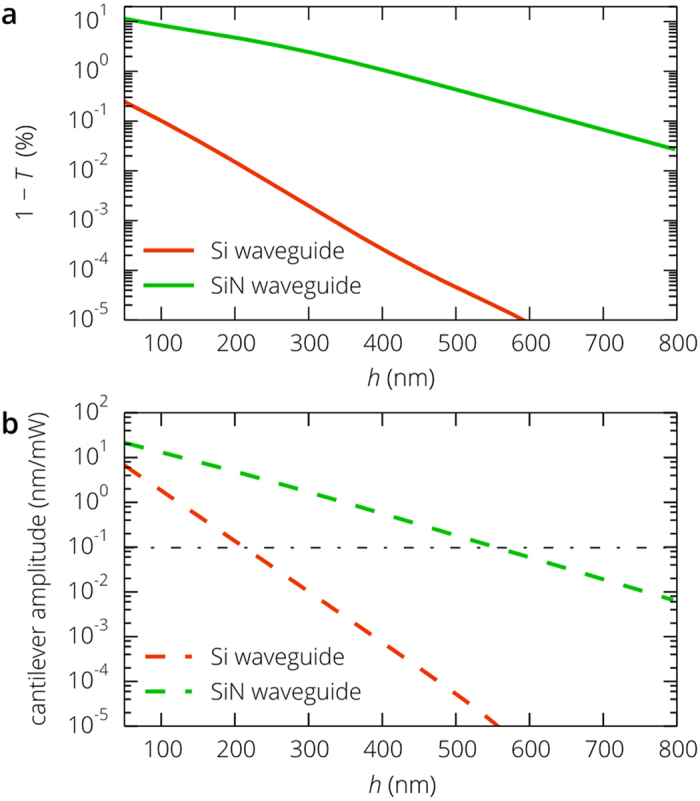
Properties of the transduction and action scheme based on the SiN waveguide. (**a**) Transmittance of the probe optical signal through the waveguide section with the SiN cantilever for the Si and SiN waveguides at a light wavelength of 1.55 μm. (**b**) Amplitude of the cantilever mechanical oscillations, induced by the fully modulated pump optical signal at a light wavelength of 1.31 μm, versus the distance to the waveguide surface. The dash-dotted line corresponds to the r.m.s. thermal vibration amplitude (0.097 nm) in the case of the 1 mW pump signal.

In contrast to silicon, silicon nitride exhibits much higher chemical stability, while still ensuring compatibility with the CMOS fabrication process. Refractive index of SiN is 1.75 times smaller than that of silicon and the 200 nm thick SiN waveguide provides poorer mode confinement. Penetration depth of the guided TE_0_ mode into air is 220 nm compared to 96 nm in the case of the silicon waveguide. Such mode size expansion is accompanied by more uniform field distribution and the normalized electric field at the position of the cantilever is significantly higher than that for the silicon waveguide (Fig. 4). This results in a strong interaction between the nanobeam and the guided mode. At a height of 50 nm, 1 – *T* is 50 times greater than in the case of the silicon waveguide, so is the linear displacement responsivity: *R*_X_|_*h*=50nm_ = 0.79 μm^−1^ corresponding to the relative intensity noise at the photodetector *RIN*_X_ = –125 dB/Hz. This feature favors the use of relatively noisy low cost laser diodes. Also important is that the responsivity decreases quite slowly as the distance between the cantilever and the waveguide increases and it is greater than 0.01 μm^−1^ at a height of 650 nm. Very similar trend is observed in the dependence of the actuation force on the cantilever position. The amplitude of oscillations induced by the fully modulated 1 mW pump signal is about 22 nm at *h* = 50 nm, which is more than one order of magnitude greater than in the case of the end-to-end coupled nanophotonic waveguide cantilevers^31^. The amplitude of induced oscillations slowly decreases with the distance from the metal film, but remains higher than the r.m.s. thermal vibration amplitude until *h* exceeds 550 nm (Fig. 5b).

The above simulations show that the linear displacement responsivity for photonic waveguides is of the order of 1 μm^−1^ and in principle can be further increased by half an order of magnitude. However, insulator and SOI structures suffer from the fact that a significant portion of the electromagnetic field is concentrated in the waveguide core and the power flow in the substrate is higher than in the cover (air or vacuum) layer, since the penetration depth the guided mode with the wavenumber *β*_g_ into air or vacuum 
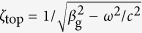
 is several times smaller than that into the substrate 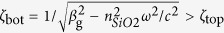. Accordingly, the normalized amplitude of the electric field at the position of the cantilever is fundamentally limited. This problem can be solved only by avoiding field localization in the waveguide core and in the substrate, which is impossible with all-dielectric waveguides. Nevertheless, surface waves can overcome this limitation if the penetration depth of the electromagnetic field into one of the media is much smaller than in the other. At optical frequencies, such a situation is realized for surface plasmon polaritons (SPPs)^45^.

SPPs being collective excitations of the conductive electron on the metal surface can be simply thought of as the propagating TM waves with the dispersion relation 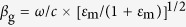 45, where *ε*_m_ is the dielectric constant of the metal. Penetration depth of the electromagnetic field into the metal is only about 20 nm and most of the energy typically flows in air. Amplitude of the electric field decreases with the distance from the metal surface as 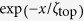, where *ζ*_top_ depends strongly on the SPP frequency. For an SPP propagating along the smooth gold^46^ or copper^47^ surface, *ζ*_top_ = 2.65 μm at λ = 1.55 μm, which is greater than the light wavelength in vacuum. However, it should be noted that in the case of the silicon photonic waveguide we are fundamentally limited in the operating wavelength of light, since silicon strongly absorbs photons with energies greater than the bandgap energy *E*_g_^Si^ = 1.1 eV (λ < 1.1 μm). Plasmonic waveguides, in turn, allow to operate at much shorter wavelengths. As λ decreases to 850 nm, *ζ*_top_ decreases to 705 nm. At the same time, ohmic losses increase as the confinement to the metal surface increases and the waveguide loss rises from 15 dB/mm at λ = 1.55 μm to 71 dB/mm at λ = 850 nm, but still remains at a relatively low level for practical applications.

**Figure 6:**
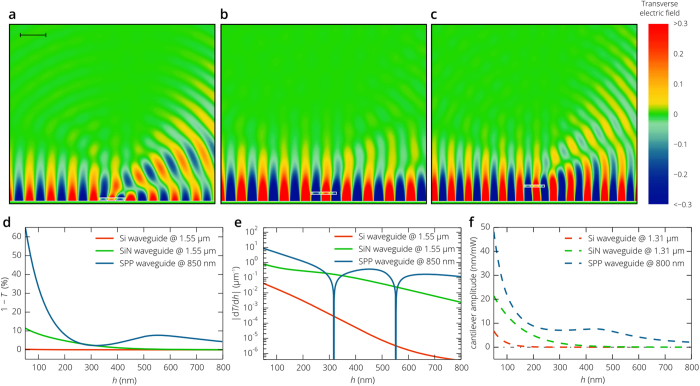
All-plasmonic biosensor. Simulated transverse electric field distribution of the SPP passing through the waveguide section with the SiN cantilever at three different positions of the cantilever: *h* = 40 nm (panel a), *h* = 280 nm (panel b), *h* = 560 nm (panel c). Panels d and e show the dependences of the transmittance and its derivative d*T*/d*h* on the distance between the cantilever and the waveguide. As opposed to the Si and SiN photonic waveguides (Fig. 5a), (1– T) does not steadily decrease with *h* increases and well pronounced minima and maxima are observed. These extrema give zero linear displacement responsivity |d*T*/d*h*| at *h *= 317 nm and *h *= 552 nm (see panel e). Panel f presents the amplitude of the cantilever oscillations induced by the fully modulated pump signal per unit power of the pump signal before it is modulated.

Figure 6a–c presents the simulated transverse electric field of the all-plasmonic gas and biosensor based on the SPP waveguide and SiN cantilever, suspended above the waveguide. In contrast to the Si and SiN photonic waveguides at telecom wavelengths, the penetration depth of the SPP field into air or vacuum (*ζ*_top_ = 705 nm) is comparable with the light wavelength (λ = 850 nm) and the profile of the nanobeam provides high overlap between the guided mode and radiation modes of the plasmonic waveguides, which eventually results in the characteristic minima and maxima in the dependence of the transmission coefficient *T* on the distance from the metal surface (Fig. 6d). At a height of 50 nm, transmission is less than 50%, 1 – *T* = 0.66, which is six times greater than in the case of the SiN waveguide and 270 times greater than in the case of the Si waveguide (Fig. 5a). In spite of the fact that the penetration depth of the SPP *ζ*_top_ = 705 nm is more than seven times larger than that of the TE_0_ mode of the silicon waveguide (*ζ*_top_ = 96 nm), 1 – *T* and |*R*_X_| decreases with the distance from the waveguide surface roughly at a similar rate (Fig. 6d,e) due to resonant coupling between guided and radiation modes of the SPP waveguide^40^, which gives extrema in Fig. 6d at a distance of 317 nm and 552 nm. In these positions of the cantilever, the linear displacement responsivity is zero and the local maxima are reached at *h* = 440 nm and *h* = 680 nm. But, obviously, the highest responsivity is achieved near the metal surface. Thus, for example, at a height of 50 nm, |*R*_X_| = 8 μm^−1^ and corresponds to the relative intensity noise at a photodetector of –104 dB/Hz, which is several orders of magnitude higher than the RIN of low cost semiconductor diodes. The responsivity can be easily further improved by operating at shorter optical wavelengths, but this is unavoidably accompanied by increase in the waveguide loss and the practical limit is dictated by the size and complexity of the whole photonic scheme. As opposed to the responsivity, optical forces are determined only by the field gradient of the guided mode and, at *h *= 50 nm, the amplitude of mechanical oscillations for the cantilever suspended above the plasmonic waveguide is only twice as large as that in the case of the SiN waveguide and seven times greater than in the case of the Si waveguide. However, since the amplitude of electric field decreases with the distance from the waveguide surface slower than for the Si and SiN photonic waveguide (Fig. 4), the amplitude of the cantilever oscillations induced by the fully modulated pump signal with a power of 1 mW surpasses, by more than one order of magnitude, the r.m.s. thermal noise amplitude even at a distance between the nanobeam and the waveguide of greater than 1 μm. At the same time, the responsivity at *h* = 1 μm is equal to 0.06 μm^−1^ and is sufficiently high for practical applications. Thus, the proposed all-plasmonic biosensor demonstrates the highest linear displacement responsivity, the most efficient optical actuation and can operate in a wide range of distances between the nanobeam cantilever and plasmonic waveguide.

### Operation in atmosphere

In vacuum, the energy of the oscillating cantilever is dissipated due to internal friction and the quality factor of the resonance is of about *Q*_int _= 3500. This gives the possibility to efficiently excite the cantilever (see equation (6)) and detect changes in mass with single-molecule resolution (see equation (8)). However, for practical applications, operation at atmospheric pressure is desirable, but, as the gas pressure increases, the hydrodynamic drag becomes dominant over the internal dissipation significantly decreasing the quality factor of the mechanical resonator. Firstly, damping is caused by individual gas molecules, which collide with the oscillating cantilever. The corresponding quality factor can be given by^48^


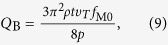


where *p* is the gas pressure and *υ*_*T*_ is the average thermal velocity of gas molecules. At room temperature in air at a pressure of 1 atm *Q*_B_ = 26.8, which is more than two orders of magnitude lower than the quality factor limited by internal dissipation effects. Secondly, at high pressure, the density of gas is so large that we cannot neglect interaction between gas molecules and viscous effects must be considered. The hydrodynamic drag force due to viscous friction of the oscillating cantilever with the dense gas per unit length of the cantilever can be written as 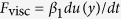, where *u*(*y*) is the displacement of the cantilever from the equilibrium position at the distance *y* from the fixed end and *β*_1_ is the damping constant, which can be estimated as^49^


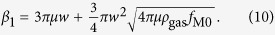


In this expression, *μ* is the dynamic viscosity and *ρ*_gas_ is the density of the gas. Accordingly, the quality factor associated with the viscous friction is equal to


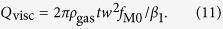


**Figure 7:**
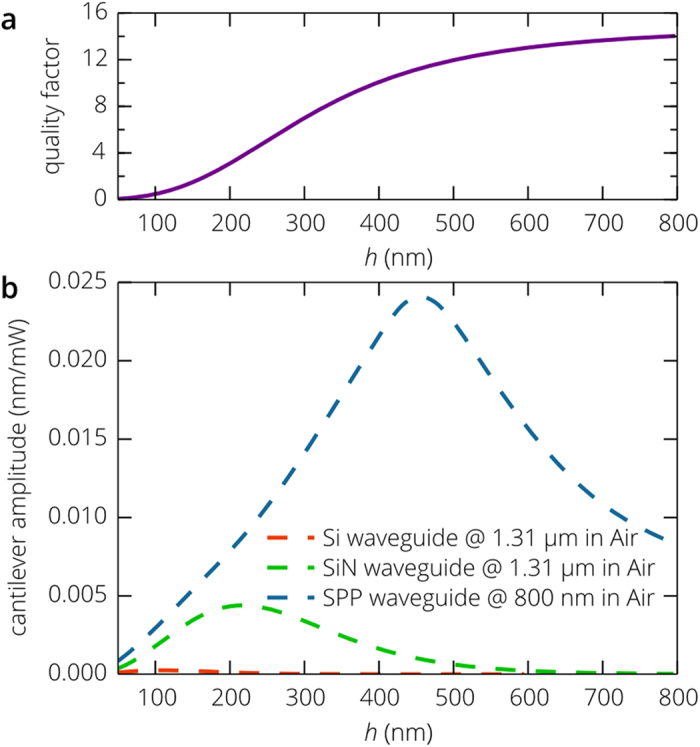
Operation of the all-nanophotonic biosensor in atmosphere. (**a**) Dependence of the quality factor of the mechanical resonator on the cantilever position above the nanophotonic waveguide in atmosphere. (**b**) Amplitude of mechanical oscillations induced by the fully modulated pump optical signal as a function of the separation distance between the SiN cantilever in air and the waveguide surface.

Finally, since the cantilever is placed above the substrate at a distance of several tens of nanometers (Fig. 1), an additional drag force appears due to the small gaps between the beam and the waveguide and between the beam and the SiO_2_ surface. The vibrating beam squeezes the gas film between the beam and substrate and causes the gas to flow towards the beam edges. Following Ref. [49], we can roughly estimate the squeeze-film quality factor as


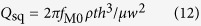


and calculate the total quality factor as 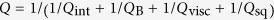. Figure 7a shows that the damping force becomes very strong, when the cantilever is separated from the substrate by a distance of a few nanometers and *Q* becomes much smaller than unity. As the cantilever is moved away from the waveguide, the quality factor steadily increases and, at a distance of 1 μm, the gap between the waveguide and the SiN beam does not have any impact on cantilever damping and *Q* ≈ 1/(1/*Q*_B_ + 1/*Q*_visc_) = 15.

In contrast to the case of induced oscillations in vacuum (Fig. 6f), the vibration amplitude, being proportional to the quality factor, does not monotonically decrease as *h* increases (Fig. 7b) and for all three waveguides the optimal positions of the SiN beam above the waveguide are clearly observed. Silicon waveguide provides the best confinement of the optical mode to the waveguide core and the highest gradient of the electromagnetic field becomes practically useless at atmospheric pressure: the maximum amplitude is achieved at *h* = 110 nm and is equal to only 2 × 10^−4 ^nm per milliwatt of the pump optical signal, while *R*_X_ is less than 0.015 μm^−1^ and *Q* = 0.6. SiN waveguide demonstrates much better characteristics: the amplitude exceeds 4 × 10^−3^ nm/mW for *h* in the range from 200 nm to 300 nm, which combined with the high linear displacement responsivity gives a sensitivity per power of the pump signal of (2 – 4) × 10^5^ Da∙mW at a measurement bandwidth of 100 Hz . In order to improve the sensitivity, one needs to operate with higher mechanical quality factors. Due to the squeeze-film damping, this can be possible only by moving the cantilever away from the waveguide, but as *h* increases, both the displacement responsivity and the oscillation amplitude rapidly decrease and the best sensitivity for the considered SiN waveguide is achieved at a distance of 140 nm from the waveguide surface, where the quality factor is about unity. However, this mass sensitivity is associated with the inherent properties of the waveguide and the cantilever in air and the RIN at the photodetector will be limited by the laser, as follows from Eq. (8), unless the laser noise is about –180 dB/Hz.

As opposed to photonic modes of dielectric waveguides, the linear displacement responsivity and the optical force acting on the cantilever are not monotonically decreasing functions of the gap distance between the waveguide and the cantilever (Fig. 6e,f). They exhibit maxima at *h* ≈ 440 nm giving a possibility to operate with a mechanical quality factor of about 11. In addition, relatively high penetration depth of the electromagnetic field into air and high field confinement in the air region above the metal surface (for details see section *Improvement of the all-nanophotonic gas and biosensor*) result in a high magnitude of these maxima, so that the maximum sensitivity of the biosensor per power of the pump signal is equal to 6.5 × 10^4^ Da∙mW at a measurement bandwidth of 100 Hz. In addition, this is achieved at the moderate mechanical quality factor diminishing the influence of the laser noise on the sensitivity of the proposed biosensor and giving a possibility to use freely lasers with the RIN of up to –145 dB/Hz without reduction in accuracy of mass detection.

## Discussion

We have proposed a novel scheme of highly integrated NEMS biosensor with all-nanophotonic transduction and actuation. Such a configuration consists of the nanophotonic waveguide and the nanobeam cantilever placed above it at a small distance (Fig. 1), which can be controlled with sub-nanometer accuracy in the manufacturing process using wet chemical etching. This ensures high fabrication reproducibility making the proposed biosensor an ideal candidate for parallel monitoring and mass production. Mechanical vibrations of the cantilever are excited by the sinusoidally modulated pump optical signal propagating along the waveguide and the same waveguide serves as an optical transducer: the vibrating cantilever controls the transmission of the continuous wave probe optical signal through the section with the suspended nanobeam. Such an optical transduction scheme does not use interferometric effects and therefore low noise incoherent light sources with relatively broad emission spectrum can be utilized.

The proposed all-nanophotonic scheme demonstrates high transduction and actuation efficiency even in the case of the nanobeam cantilever with a small beam cross-section of 0.09 μm^2^. At telecom wavelengths, the amplitude of mechanical oscillations *X*_A_ excited by the fully modulated 100 μW pump signal propagating along the Si photonic waveguide exceeds 0.7 nm, while the displacement responsivity *R*_X_ of the read-out scheme approaches 0.04 μm^−1^, which is significantly higher than that of the optical fiber based systems. Despite that these numbers can be further improved by optimizing the waveguide geometry, a higher performance is achieved with SiN photonic waveguides. For the same waveguide dimensions, *R*_X_ increases by more than one order of magnitude (up to 0.8 μm^−1^) and *X*_A_ reaches 2.2 nm. It should be noted, however, that both *X*_A_ and *R*_X_ rapidly decrease as the distance between the cantilever and the waveguide increases (Fig. 5) and one has to place the cantilever close to the waveguide surface. For this reason, the operation at short wavelengths and a strong mode confinement is not reasonable. The above problem can be overcome by using plasmonic waveguides, which provide stronger interaction between the cantilever and the guided mode (for details see section *Improvement of the all-nanophotonic gas and biosensor*). At *λ *= 850 nm, which is chosen because of the poor SPP confinement at telecom wavelengths, *R*_X_ is about 9 μm^−1^ for the cantilever in the proximity of the waveguide surface and is equal to 0.17 μm^−1^ for the cantilever suspended at a distance of 660 nm above the SPP waveguide. Thanks to the high responsivity and efficient actuation in vacuum, the proposed nanophotonic transduction schemes do not affect the mass-detection sensitivity of the biosensor, which is limited by the thermomechanical noise of the cantilever rather than the properties of the optical source. At a measurement bandwidth of Δ*f* = 100 Hz and a pump signal power of *P* = 200 μW, δ*m* is about 6.5 kDa for the SiN waveguide and about 2.9 kDa for the SPP waveguide. This makes both schemes capable of single-molecule resolution. The sensitivity can be further improved by increasing the measurement bandwidth (i.e., increasing the measurement time), as can be seen from equation (8).

In atmosphere, NEMS are difficult to operate due the very low quality factor caused by hydrodynamic drag. Despite that the SPP waveguide and SiN photonic waveguide demonstrate comparable performance in vacuum, squeeze-film damping drastically limits the cantilever actuation efficiency at small air gaps between the waveguide and cantilever, and photonic schemes show relatively low sensitivity. On the other hand, we have shown that conventional low noise lasers do not limit the sensitivity of the plasmonic scheme (which is still determined by inherent mechanical properties of the cantilever) thanks to the ability to operate efficiently at relatively large separation distances between the waveguide and cantilever. The resolution limit δ*m* approaches 65 kDa at Δ*f* = 100 Hz and *P* = 1 mW, which is small enough to detect a single biomolecule.

To conclude, the proposed biosensor scheme is easily manufactured, extremely compact and compatible with standard CMOS fabrication processes. This, combined with the high sensitivity, forms the backbone for parallel monitoring and analysis with a single-molecule resolution at the chip scale.

## Methods

Transmission of the probe and pump optical signals through the waveguide section with the cantilever was accurately simulated using the 2D finite element method in COMSOL Multiphysics. For the schemes based on the silicon and silicon nitride waveguides, the simulation domain was set to 25 μm in length and 14 μm in height and was surrounded by the perfectly matching layer. The mesh size did not exceed 20 nm near the waveguide and cantilever and was about 80 nm near the top and bottom boundaries of the simulated region. In case of the plasmonic waveguide, the simulation domain was 25 μm × 7.1 μm and the mesh size was reduced down to 4 nm near the cantilever and the metal surface and was equal to 2.5 nm in the metal.

## Additional Information

**How to cite this article**: Fedyanin, D. Y. and Stebunov, Y. V. All-nanophotonic NEMS biosensor on a chip. *Sci. Rep.* **5**, 10968; doi: 10.1038/srep10968 (2015).
